# Magnetic, Antiferroelectric-like Behavior and Resistance Switching Properties in BiFeO_3_-CaMnO_3_ Polycrystalline Thin Films

**DOI:** 10.3390/ma16237392

**Published:** 2023-11-28

**Authors:** Abdelilah Lahmar, Jacem Zidani, Jamal Belhadi, Ilham Hamdi Alaoui, Hussam Musleh, Jehad Asad, Naji Al Dahoudi, Mimoun El Marssi

**Affiliations:** 1 Condensed Matter Physics Laboratory (LPMC), University of Picardie Jules Verne, 33 Rue Saint Leu, 80000 Amines, France; jacem.zidani@etud.u-picardie.fr (J.Z.); jamal.belahdi@u-picardie.fr (J.B.); ilham.hamdi.alaoui@u-picardie.fr (I.H.A.); mimoun.elmarssi@u-picardie.fr (M.E.M.); 2 Physics Department, Al Azhar University-Gaza, Gaza P.O. Box 1277, Palestine; drhussmu@gmail.com (H.M.); asaadjihad@gmail.com (J.A.); najialdahoudi@gmail.com (N.A.D.)

**Keywords:** ferromagnetic, antiferroelectric-like, polycrystalline films, BiFeO_3_-CaMnO_3_, resistance switching

## Abstract

The effect of ferromagnetic CaMnO_3_ (CMO) addition to structural, magnetic, dielectric, and ferroelectric properties of BiFeO_3_ is presented. X-ray diffraction and Raman investigation allowed the identification of a single pseudocubic perovskite structure. The magnetic measurement showed that the prepared films exhibit a ferromagnetic behavior at a low temperature with both coercive field and remnant magnetization increased with increasing CMO content. However, a deterioration of magnetization was observed at room temperature. Ferroelectric study revealed an antiferroelectric-like behavior with a pinched *P*–*E* hysteresis loop for 5% CMO doping BFO, resulting in low remnant polarization and double hysteresis loops. Whereas, high remnant polarization and coercive field with a likely square hysteresis loop are obtained for 10% CMO addition. Furthermore, a bipolar resistive switching behavior with a threshold voltage of about 1.8 V is observed for high doped film that can be linked to the ferroelectric polarization switching.

## 1. Introduction

Interest in multiferroic materials has been steadily growing due to their interlinked electric, magnetic, and structural order parameters, resulting in concurrent ferromagnetic, ferroelectric, and magnetoelectric characteristics [1,2,3]. Among these materials, BiFeO_3_ (BFO), which has been extensively investigated, exhibits ferroelectric behavior with a Curie temperature (TC) of 1083 K and G-type antiferromagnetic properties with a (TN) of 643 K [4,5]. Furthermore, it maintains a distorted rhombohedral structure within the (*R3c*) space group at room temperature, rendering it an appealing option for numerous technological applications [6,7]. A review of the literature has revealed that bulk BFO is susceptible to high leakage currents attributed to defects within its matrix [8,9]. However, BFO thin films have garnered increased attention due to reported enhancements in remnant polarization (Pr) and magnetization when compared to bulk single crystal [10]. Nevertheless, the low resistivity of BFO films can impede polarization switching, representing a significant concern primarily due to the complex defect chemistry [11,12,13]. It is important to note that reducing the grain size and appropriate doping can effectively mitigate leakage currents in BFO, improving its microstructure. Extensive research has been conducted to enhance the electrical and magnetic properties of BFO through doping strategies [8,14,15,16,17,18,19,20,21,22].

It is worth noting that significant progress has been made in showcasing the multifunctionality of BFO-based materials. Notably, researchers have reported intriguing enhancements in multiferroic properties in both epitaxial and polycrystalline BFO-based thin films. For instance, the incorporation of rare-earth elements into epitaxial BFO thin films has led to the emergence of a morphotropic phase boundary (MPB) between the rhombohedral phase (*R3c*) and orthorhombic phase (*Pnma*) at a critical composition of x = 0.14, particularly for elements such as Gd and Sm. In this composition range, distinctive features such as pinched hysteresis loops with small remnant polarization have been documented, indicating the presence of antiferroelectric-like behavior in these compositions [23,24]. Transmission electron microscopy investigations have provided corroborating evidence of local ordering, similar to what is observed in antiferroelectric (AFE) PbZrO_3_ materials [25]. The observation of this region holds particular promise for achieving a significant piezoelectric response. However, it is important to note that the macroscopic response of these compositions continues to exhibit primarily ferroelectric behavior.

On the other hand, the phenomenon of resistance switching (RS) in BFO-based materials has garnered significant attention, particularly in the context of nonvolatile memory devices. Y. Shuai et al. reported nonvolatile bipolar resistive switching in Au/BiFeO_3_/Pt configurations [26]. Additionally, Xinman Chen et al. documented bipolar RS behaviors in BiFeO_3_ thin films with Pt/BiFeO_3_/LNO setups [27]. The strategy of doping BFO has also emerged as a compelling avenue for enhancing RS properties. Yang C. H et al. observed such properties in Ca-doped BiFeO3 films, employing conductive atomic force microscopy [28]. Furthermore, Mi Li et al. reported RS behavior in metal/Bi_0.95_La_0.05_FeO_3_/Pt-sandwiched structures [29]. Through this brief literature review, it becomes evident that BFO-based materials continue to captivate researchers in the field of multiferroics due to their adaptability and multifunctionality. Notably, their physical and chemical properties can be readily adjusted to achieve desired characteristics.

Prior studies have demonstrated that the incorporation of RMnO_3_ (where R represents a rare-earth element) into BFO thin films can effectively enhance both ferroelectric and magnetic properties, even at doping concentrations of up to 10% [30]. These investigations have revealed that even a minor doping concentration can instigate significant structural changes, characterized by the emergence of Jahn–Teller distortion. This structural transformation has been ascribed to the presence of Mn^3+^ ions within the BFO matrix [31]. However, it is noteworthy that the substitution of Bi^3+^ ions, which are stereochemically active lone pair ions, with lanthanide elements, which are nonstereochemically active ions, appears to lead to a reduction in the stereochemical activity of Bi–O bonds.

In pursuit of exploring additional functionalities and advancing our research on the impact of manganates on the physico-chemical properties of BFO polycrystalline films, our present study focuses on the influence of the manganite compound CaMnO_3_. In our previous research, we extensively discussed the presence of Mn^3+^ ions occupying Fe^3+^ sites, demonstrating its critical role in maintaining a polarization switching behavior [30,31]. Conversely, the incorporation of Ca^2+^ ions into the Bi^3+^ sites has been noted as particularly intriguing for inducing a resistance switching behavior [32,33,34]. Additionally, Lu Liu et al. reported the coexistence of unipolar and bipolar resistive switching in Bi_0.8_Ca_0.2_FeO_3_ polycrystalline thin films [32]. In this work, we place emphasis on the fabrication of BiFeO_3_-xCaMnO_3_ (Bi_1−x_Ca_x_Fe_1−x_Mn_x_O_3_ (0 ≤ x ≤ 0.1)) polycrystalline thin films and the comprehensive investigation of their structural, magnetic, and electrical properties. Remarkably, our findings reveal the presence of an antiferroelectric-like characteristic within our system, coexisting with a resistance switching behavior. This discovery holds significant promise for distinguishing between ferroresistive effects [9] and trapping phenomena [35].

## 2. Materials and Methods

Thin films were synthesized through a spin-coating process onto a commercial (111)-Pt/Ti/SiO_2_/Si substrate heterostructure. The initial materials used were Bi-acetates and Sr acetates tetrahydrate (sourced from Alpha easer, Thermo Scientific Chemicals, Karlsruhe, Germany), as well as Mn, Fe, acetylacetonate (obtained from Sigma-Aldrich, Darmstadt, Germany). These compounds were dissolved in a mixture of propionic acid and 2-methoxyethanol in a volume ratio of 1:2, resulting in a sol concentration of 0.22 mol/L. Pyrolysis of the sol-gel was carried out on a hot plate set at 260 °C. Subsequently, a final annealing process was conducted within a preheated tube furnace under a saturated oxygen atmosphere at 650 °C for a duration of 60 min. The thickness of the resulting films after annealing was determined using scanning electron microscopy (SEM) on cross-sections. Microstructural analysis of the prepared samples was performed using an Environmental Quanta 200 FEG (FEI company, Hillsboro, Oregon, USA) microscopy (SEM) operating at 5 kV. The thin films obtained in this study underwent several characterization techniques. Firstly, X-ray diffraction analysis was carried out at room temperature using a four-circle high-resolution D8 Discover Bruker diffractometer equipped with a Göbel mirror. Cu Kα radiation with a wavelength (ƛ) of 1.5418 Å was utilized for this analysis. Additionally, Raman spectroscopy was performed in a back-scattering configuration using a micro-Raman Renishaw spectrometer, with a green laser excitation source emitting at 514.5 nm. The laser power was carefully maintained below 20 mW to prevent any sample heating effects. For the assessment of magnetic properties, a commercial Physical Property Measurement System (PPMS DynaCool, Quantum Design) was employed. Magnetization measurements (*M*(*T*)) were conducted at both 2 K and 300 K, with an applied magnetic field up to *H* = 1 kOe. Dielectric measurements were carried out as a function of temperature and over a frequency range spanning from 100 Hz to 1 MHz. These measurements were performed using a Solartron Impedance/GAIN-PHASE analyzer SI-1260 (AMETEK scientific instruments, Oak Ridge, TN, USA), with a probing AC electric field amplitude of 100 mV. Ferroelectric investigations were conducted by measuring the polarization–electric field (*P*–*E*) hysteresis loops at a frequency of 10 kHz. A TF Analyzer 3000, aix-ACCT system, was employed for this purpose. To assess the leakage current properties, a Keithley 2611A source was utilized. All electrical measurements were executed using a metal–dielectric–metal geometry, with sputtered-Pt circular top electrodes featuring diameters of 250 µm. These electrodes were deposited through a shadow mask to ensure precise placement.

## 3. Results

### 3.1. Microstructural and Structural Investigation

**Figure 1:**
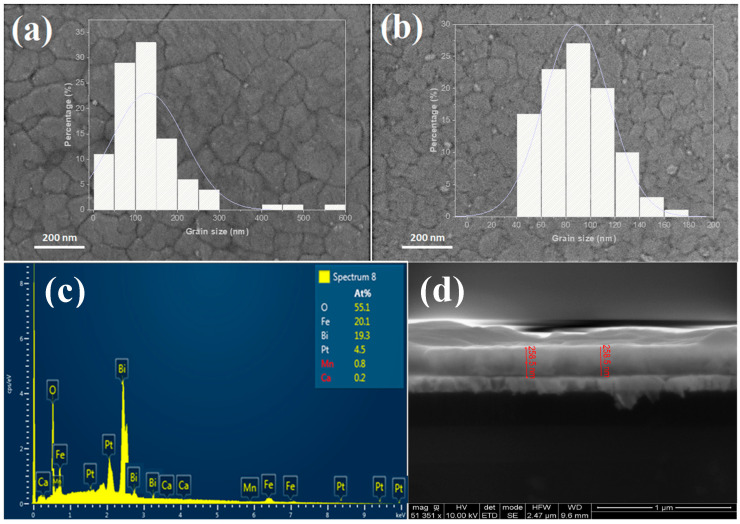
Surface morphologies of studied specimens: (**a**) 5 CaBFOM; (**b**) 10 CaBFOM; (**c**) example of EDX analysis for 10 CaBFOM film; and (**d**) example of cross-section for 10 CaBFOM film.

Figure 1a,b present the scanning electron microscopy (SEM) images of the prepared thin films. In the case of the 5 CaBFOM phase, the microstructure reveals plate-like grains, with distinct grain boundaries that are readily visible. It is worth noting that as the concentration of doping elements increases, it results in the fragmentation of larger grains, leading to a noticeable reduction in grain size. The average grain sizes were measured to be 130 nm and 89 nm for the 5 CaBFOM and 10 CaBFOM thin films, respectively. It seems that the increasing of the CMO concentration inhibits the grain growth while sintering.

**Figure 2:**
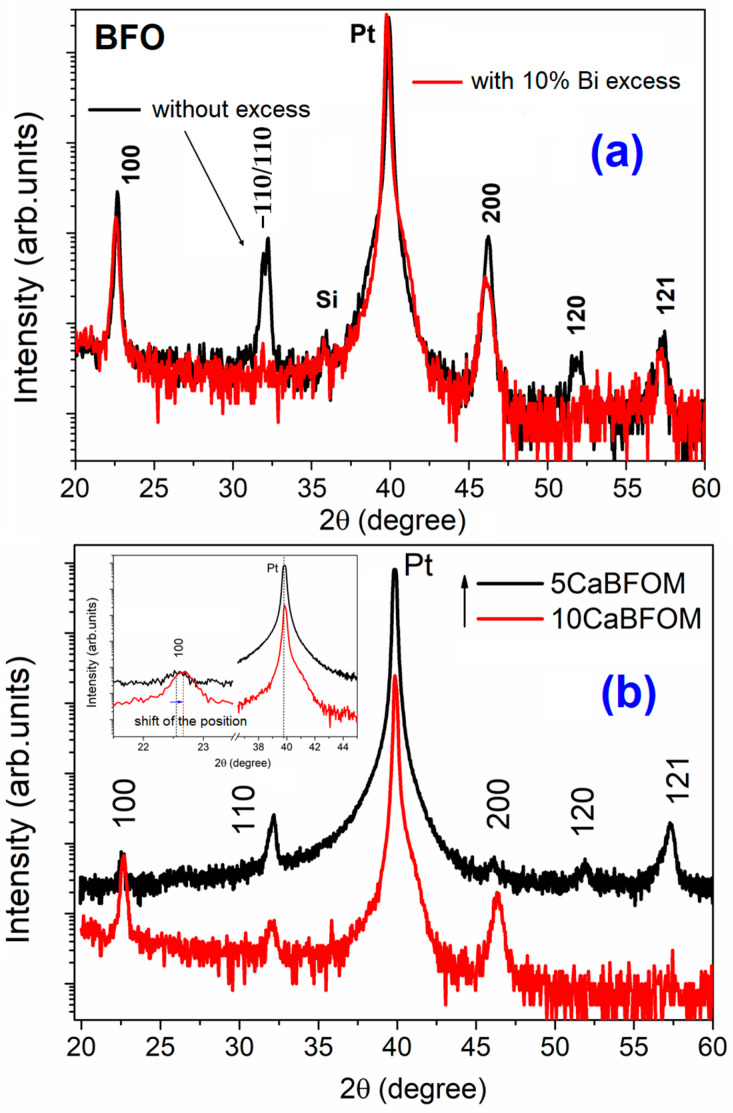
XRD pattern of (**a**) BFO thin films with and without Bi excess; (**b**) comparison of 5 CaBFOM and 10 CaBFOM thin films. All films are deposited on (111)-Pt/Ti/SiO_2_/Si. Pt and Si denote peaks belonging to the substrate. The reflections are indexed according to a pseudocubic unit cell.

The EDX spectra carried out on both films showed the expected starting elements Bi, Fe, Ca, Mn, and O. Figure 1c shows an example of the EDX analysis carried out on 10 CaBFOM film. The average thickness of the prepared films was found to be approximately 280 nm (see Figure 1d as example). Figure 2 illustrates the X-ray diffraction (XRD) patterns of the films under investigation, with the reflections indexed based on a pseudocubic unit cell. All films exhibited pure perovskite phases, and these findings are considered accurate within the precision of the employed device. It is indeed challenging to discern detailed structural changes when transitioning from a 5% to a 10% CaMnO_3_ concentration. Nevertheless, a noticeable trend emerges in favor of orientation in the (h00) direction when comparing the diffraction patterns. Remarkably, the splitting reflections (110) and (−110), which are observed in pure BFO with excess (Figure 2a), were not detected upon using 10% Bi excess or doping with CaMnO_3_, as shown in Figure 2b. Instead, they appear to have merged into a single peak around 32° (2θ), consistent with previous reports that have linked this phenomenon to structural transformations [30,31]. Additionally, upon closer examination of the position of the (100) reflection (as depicted in the inset), a clear shift towards higher angles is evident with an increase in the CaMnO_3_ concentration to 10%. The lattice spacing, determined from the (100) pseudocubic peaks (d_100_ values), closely approximates the in-plane lattice spacing of Pt (100), which is 0.3923 nm. This alignment suggests a good lattice match between the prepared thin films and the Pt-crystal substrate as a possible contributing factor to these observations.

**Figure 3:**
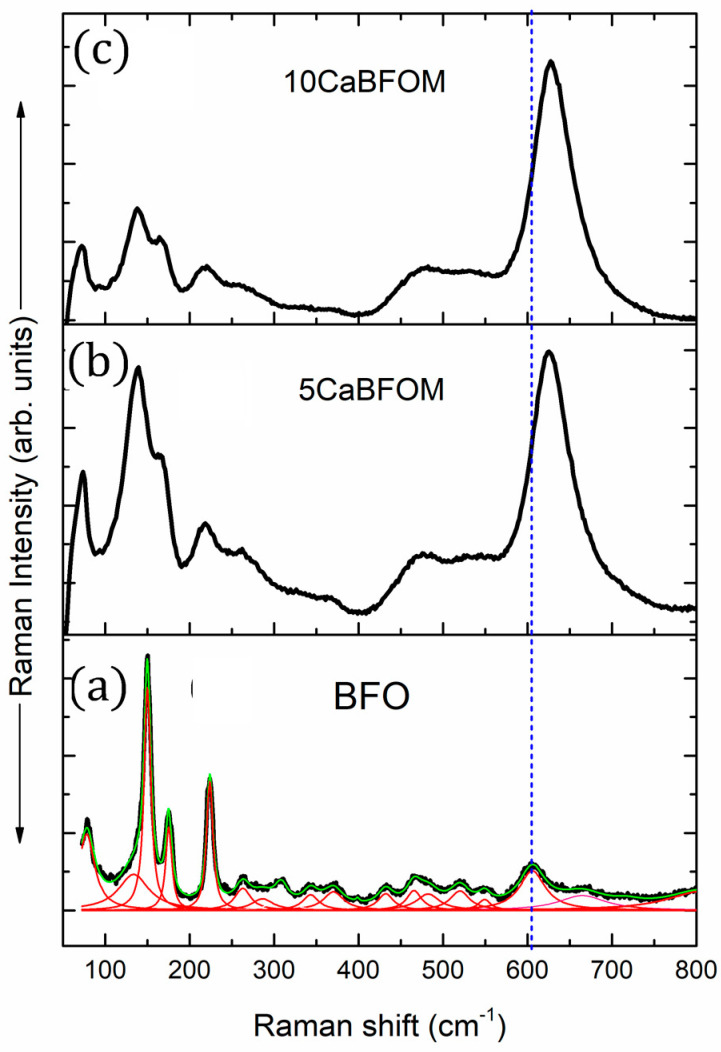
Raman spectra of the investigated thin films: (**a**) BFO for comparison, (**b**) 5 CaBFOM, and (**c**) 10 CaBFOM. The dotted line shows the shift of the mode with substitution.

Raman spectroscopy was employed to gain further structural insights not accessible through X-ray diffraction (XRD). Figure 3 displays the Raman spectra of both studied films in comparison with that of the pure BFO thin film.

Recall that in BFO bulk materials, the selection rules for the Raman active modes predicted 27, 13, and 8 modes for monoclinic, rhombohedral, and tetragonal symmetries, respectively. As depicted from the deconvolution of the Raman spectrum of BFO polycrystalline thin films, 18 vibration modes are founded, suggesting the monoclinic symmetry as reported by Kartopu et al. [36]. The assignment of each mode can also be found in this reference, noting that all observed modes below 300 cm^−1^ were attributed to Bi–O covalent bonds. The substitution of Bi^3+^ by Ca^2+^ induced the decrease in the Bi–O, and all associated vibration modes become progressively weak. Additionally, two broad bands appear around 620 cm^−1^ and within the range of 450 to 550 cm^−1^, corresponding to symmetric and antisymmetric stretching modes, respectively. In comparison to pure BiFeO_3_ (the parent phase), the presence of these bands is associated with the basal oxygen ions of the Mn^3+^O_6_ octahedra, which are linked to Jahn–Teller (JT) distortion with apparition of orthorhombic symmetry. Further discussion regarding the development of these features when doping BFO with rare-earth manganite can be found in our prior research [30,31,36,37]. For instance, Ca-doped BiFeO_3_ bulk materials are successfully refined by assuming a mixture of the rhombohedral *R3c* and orthorhombic Pnma space groups in the work of Sunil Chauhan et al. [38]. Further, the authors reported that the contribution of orthorhombic phase increases with increasing Ca content in BiFeO_3_ nanoparticles. Upon a closer look at obtained Raman spectra in the present work, it seems that the intensity ratio of the peak at 150 cm^−1^ (the most intense peak in BFO spectrum) with regard to the band appearing at 627 cm^−1^ (the most intense peak in CaBFOM spectra) exceeds unity by increasing the concentration of CaMnO_3_. It is very likely that at a high concentration, the structure changes from orthorhombic to tetragonal symmetry. Such a behavior was also observed in the BFO-LaMnO_3_ system [31].

### 3.2. Magnetic Investigations

**Figure 4:**
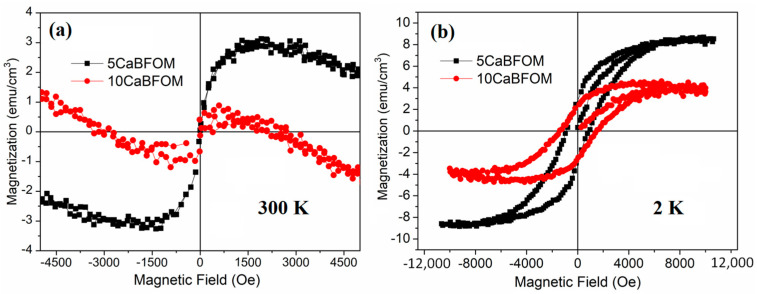
Magnetic hysteresis loop for the investigated thin films (**a**) at 300 K; (**b**) at 2 K.

The magnetic hysteresis curves for the studied specimens at both room temperature (RT) and 2 K are presented in Figure 4. It is worth noting that CaMnO_3_ is recognized as an antiferromagnetic insulator (AFMI) material, characterized by the presence of both four- and tri-valent manganese ions [39]. This manganite exhibits a complex magnetic behavior at low temperatures, transitioning from antiferromagnetic (G-type AFM) to paramagnetic ordering with a Neel temperature around 125 K and eventually revealing a weak ferromagnetic component below 10 K [40,41,42].

The strong G-type antiferromagnetic (AFM) order observed at 120–125 K in CaMnO_3_ is attributed to superexchange interactions between Mn^3+^ and Mn^4+^ ions. Conversely, the ferromagnetic (FM) order in CMO arises from the presence of double exchange interactions [43,44]. In the present study, as depicted in Figure 4a, there is no significant improvement in magnetization observed at room temperature when compared to what is observed for the BFO thin film [9]. However, a decrease in magnetic properties is evident when increasing the CMO content from 5% to 10% at room temperature. Similar behavior was reported with the addition of LaMn_0.5_Co_0.5_O_3_ to BFO thin films [45]. Interestingly, the influence of CMO addition on magnetization becomes more pronounced at low temperatures. As shown in Figure 4b, clear ferromagnetic hysteresis is observed for both investigated films. Notably, the increase in CMO content results in an elevation of the magnetic coercive field from 900 Oe for 5 CaBFOM to 1440 Oe for 10 CaBFOM films, but with maintaining almost the same value of remnant magnetization of 2.50 emu/cm^3^, which indicates that the material becomes magnetically hard. However, the saturation magnetization decreased by half with increasing CMO content from 5 to 10%. This behavior is plausible when considering that the increase in CMO content leads to a change in the composition, thus the increase in corresponding magnetic interactions, leading probably to the increase in the anisotropy magnetic field.

### 3.3. Dielectric and Ferroelectric Investigation

In order to investigate the impact of CaMnO_3_ doping on the dielectric and ferroelectric properties of BiFeO_3_, we conducted measurements of dielectric permittivity and polarization on both of the studied samples in comparaison with the data reported for pure BFO.

**Figure 5:**
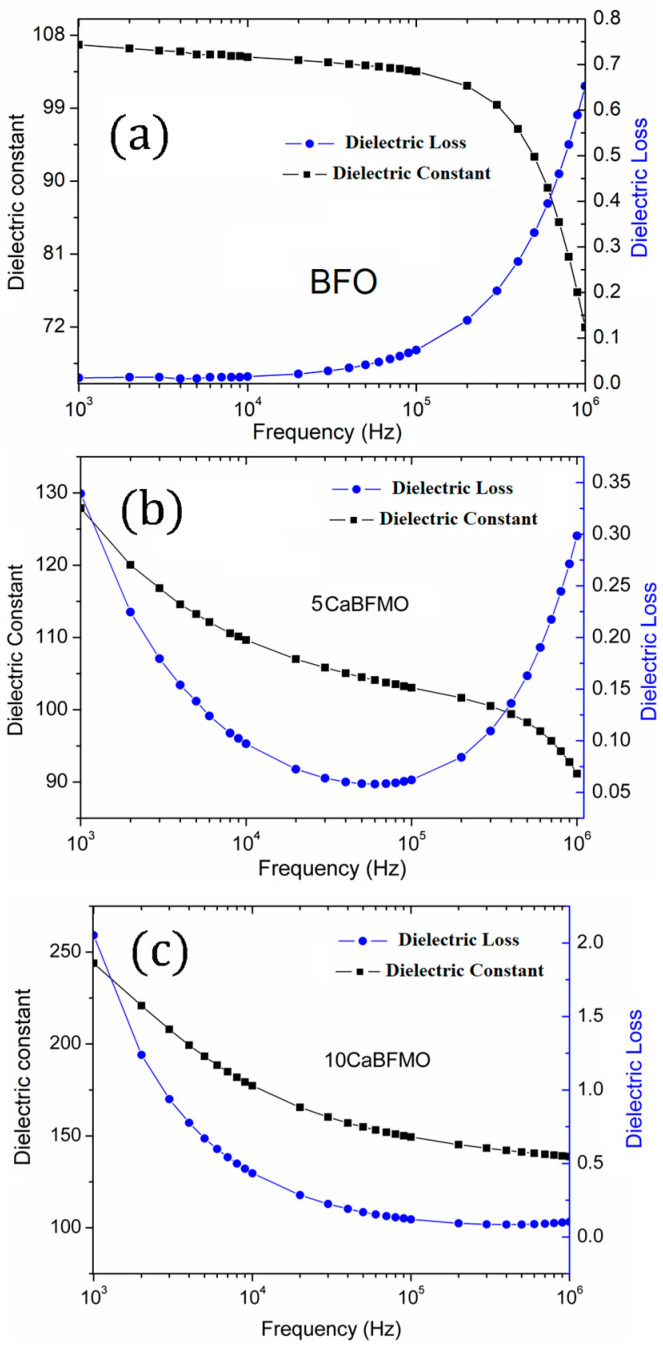
Room temperature frequency dependence of the constant dielectric and the dielectric losses for (**a**) BFO thin film, (**b**) 5 CaBFOM thin film, and (**c**) 10 CaBFOM.

In Figure 5, we present the dielectric permittivity and losses as functions of frequency at room temperature for BFO, 5 CaBFMO, and 10 CaBFMO samples. Initially, the pure BFO shows almost a linear dielectric constant around 100 until 10^5^ Hz and then drops down (Figure 5a). This behavior is due to the existence of interface states originating from the contact between the thin film and electrode during the elaboration of the BFO thin film. Indeed, during the measurement, the interfacial charge trapped is enabled to follow the alternating current variations at a high frequency, giving rise to a small dielectric constant at high frequencies. Similar behavior is reported in some Bi-based thin films [46,47]. It is also observed at the same time that the dielectric loss increases suddenly at a high frequency, which could be derived from the polarization relaxation of the inherent electric moment in BiFeO_3,_ or from the displacement polarization of different valence ions [48]. Note that the internal interfacial barrier or electrode effects have been pointed out by Shao Wei Wanga et al. [47] as a possible cause for such behavior. It is evident from Figure 5b that the 5 CaBFOM sample exhibits a consistent decrease in the dielectric constant as the frequency increases. Concurrently, the dielectric losses are notably higher at lower frequencies but decrease significantly as the frequency rises, reaching a minimum value of 0.05. These findings are indicative of space charge activation at lower frequencies, a well-known phenomenon in BFO-based materials. At the high frequency, it behaves similarly to the pure BFO with an increase in dielectric losses between 10^5^ and 10^6^ Hz. For the 10 CaBFOM thin film, and as is depicted in Figure 5c, the frequency dispersion of the dielectric constant is more pronounced because the increase in the CMO amount induced a change in the chemical gradients in the thin film, thus showing more relaxation phenomena. The dielectric loss until 10^6^ Hz seems to decrease considerably, but it is likely that the branch of increase in dielectric losses might be observed in the MHz frequency zone. Comparably, the dielectric permittivity of the 5 CaBFMO sample is approximately two times higher than that of the 10 CaBFMO sample. This difference can be attributed to domain pinning resulting from the presence of dipole defects (further discussed below).

**Figure 6:**
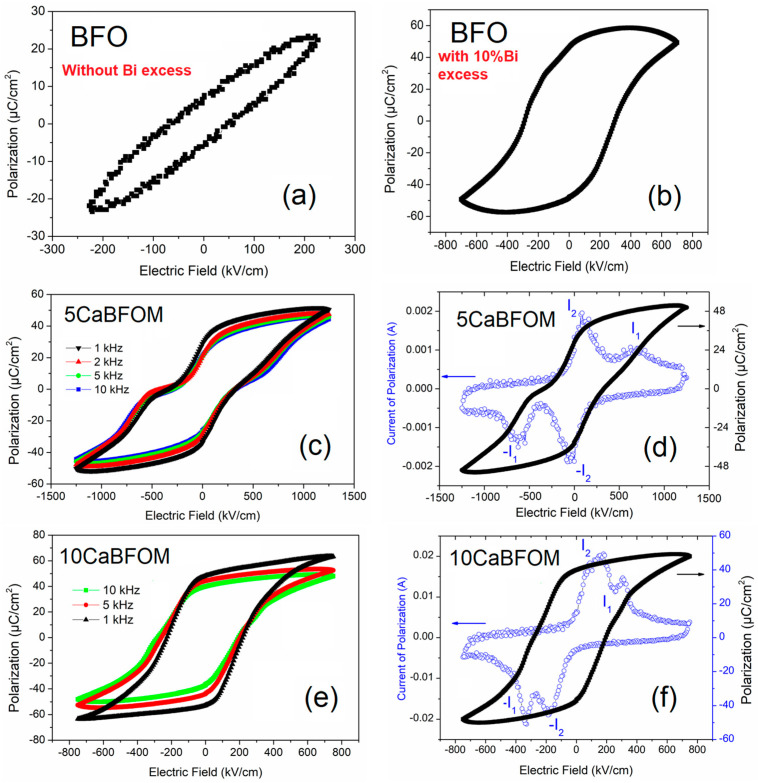
Room temperature polarization hysteresis *P*–*E* loops for (**a**,**b**) BFO without and with 10% Bi excess; (**c**) 5 CaBFMO at different frequencies; (**d**) 5 CaBFMO at 10 kHz with the corresponding polarization current switching; (**e**) 10 CaBFMO at different frequencies; and (**f**) 10 CaBFMO at 10 kHz with the corresponding polarization current switching.

Figure 6a,c showcase the ferroelectric *P*–*E* hysteresis loops measured at room temperature for the 5 CaBFMO and 10 CaBFMO films at various frequencies. The hysteresis loops of BFO with and without excess were given for comparison.

It is well known that the defect chemistry in BFO is related mostly to the volatility of Bi that created Bi vacancies. In this case, oxygen vacancies may form for compensation (using Vink–Kröger notation):

$$2{Bi}_{Bi}+3O_{O}=2V_{Bi}^{\prime \prime \prime }+3V_{Ö}+{\mathrm{Bi}}_{2}O_{3}$$

The high leakage current and conductivity hindered the switching polarization for the pure BFO, as is shown Figure 6a, noting that similar behavior is reported for some Bi-based materials [49]. However, the excess of 10% Bi may create donor states that could compensate oxygen vacancies and lead to an overall improvement of the electrical properties. In such a case, polarization switching is not hindered, as can be depicted from Figure 6b. Albeit, the *P*–*E* loop still shows some irregularity. It is worth noting that co-doping is shown to be a good strategy to improve BFO functionality [49].

Concerning CMO-modified BFO thin films, the ionization of oxygen vacancies yields electrons to compensate hole carriers created by Ca^2+^ for maintaining the highly stable Fe^3+^, as in the case of Ca-doped BiFeO_3_ [28]. Figure 6c,e show that both samples exhibit fully saturated *P*–*E* loops with a remarkably high maximum polarization exceeding 40 µC/cm^2^. It is important to note that we observe an enhancement in maximum polarization as frequency decreases. This increase can be attributed to the impact of leakage currents, which become more prominent at lower frequencies, as evidenced in the dielectric measurements. Another noteworthy observation is that the 5 CaBFMO film exhibits a clear double *P*–*E* loop. Figure 6d,f depict the *P*–*E* loops and their corresponding polarization current switching measured at 10 kHz for the 5 CaBFMO and 10 CaBFMO films, respectively. The polarization current curves for both films reveal the presence of four switching current peaks, a characteristic indicative of AFE or non-ergodic states. The observed AFE character can be attributed to the formation of various defect dipoles involving oxygen and cation vacancies, aimed at achieving charge balance within the material. In ferroelectrics, ordered defect dipoles can act as pinning centers within domains, impeding the switching of ferroelectric domains when exposed to an externally applied electric field [50,51]. It is worth noting that the electric field corresponding to the current peak I_1_, which signifies the transition from antiferroelectric-like to ferroelectric behavior (AFE–FE), is twice as high in the 5 CaBFMO film compared to the 10 CaBFMO film. This observation implies that the static electric field generated by dipole defects is stronger in the 5 CaBFMO film. This higher internal static electric field can hinder long-range polar order, resulting in a lower remanent polarization with clear antiferroelectric (AFE)-like behavior observed in the 5 CaBFMO film. This phenomenon can explain the lower dielectric permittivity observed in the 5 CaBFMO film due to increased domain pinning.

In their theoretical work, Bin Xu et al. [52] reported that the pinched loop is probable when FE and AFE are very close to each other in energy at morphotropic phase boundaries (MPBs). The authors surmise that this type of behavior occurs in the so-called hybrid improper ferroelectrics where a larger anti-polar amplitude and a small finite polarization exist simultaneously.

It is important to mention that the generation of a double hysteresis loop in ferroelectric BiFeO_3_ materials has potential applications in innovative nanodevices, including high-density multistate data storage [50]. This property opens up opportunities for exploiting the unique characteristics of these materials in advanced technological applications.

### 3.4. Resisting Switching Behavior

**Figure 7:**
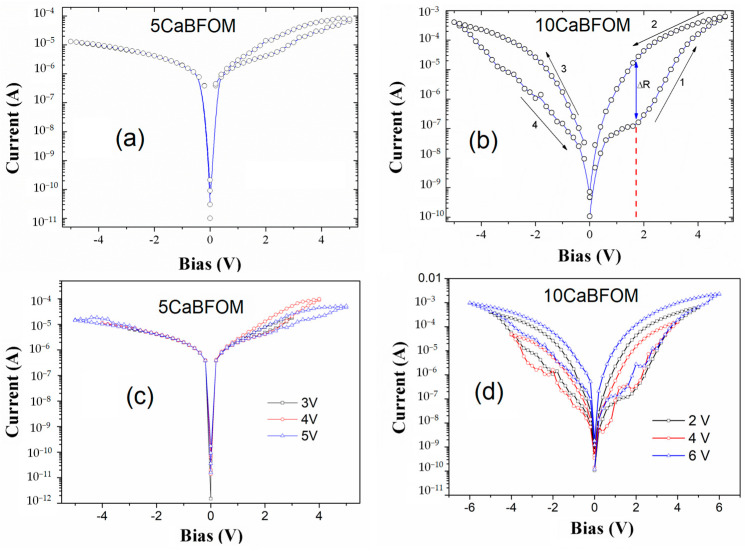
IV characteristics of (**a**) 5 Ca BFMO thin film; (**b**) 10 Ca BFMO thin film; (**c**,**d**) represent the IV at different applied biases for 5 CaBFOM and 10 CaBFOM, respectively.

Electrical measurements have revealed that all of our samples exhibit resistive switching behavior without the need for any forming process. Figure 7 displays the current–bias (IV) curve for Pt/CaBFMO/Pt films, with a maximum applied voltage of 6 V. The obtained IV curves are nearly symmetric. In the case of Pt/5 CaBFMO/Pt, shown in Figure 7a,c, a weak hysteresis is observed in the positive voltage polarity. It is important to note that this film exhibits antiferroelectric-like behavior, typically attributed to an internal bias field established by defect dipoles. These results suggest that charge carriers localized near interfaces do not contribute significantly to the resistive switching. It is known that Mn doping can significantly reduce leakage current in BFO films and enhance ferroelectric polarization. Several studies have demonstrated that ferroelectric polarization charges can control resistive switching in BFO thin films [53]. In this case, the 5% CMO content incorporated into the BFO matrix does not result in a fully saturated ferroelectric hysteresis loop and does not induce resistive switching either. Therefore, it appears that no charge-trapping effect is prominent in this film.

However, in the case of the Pt/10 CaBFMO/Pt film (Figure 7b,d), where the CMO concentration is 10%, the scenario is different.

In the case of the Pt/10 CaBFMO/Pt film, the resistive switching behavior is notable. Initially, the BFO film is in a high-resistance state, and at around 1.8 V, the film switches to a low-resistance state. The resistance ratio (ΔR) obtained for the positive bias region, as indicated by the blue arrow, demonstrates a change of approximately three orders of magnitude. The resistance ratio deduced from this figure is roughly 10^3^, which is a high value comparable to that reported in similar systems [54]. This high value positions our film as a promising candidate for nonvolatile resistive memories, as a resistance ratio of 10 is typically considered competitive with Flash technology [55,56].

It is important to note that this bipolar resistive switching, with a threshold voltage (the set tension) of about 1.8 V (indicated by the red dashed line in Figure 7b), can be directly related to the ferroelectric polarization observed in this film. However, it is worth mentioning that the current hysteresis in our case is not perfectly antisymmetric. In fact, the resistance ratio obtained in the negative bias region is smaller. This suggests that interfaces play a relatively smaller role in resistance switching compared to the impact of ferroelectric polarization in our system.

## 4. Conclusions

In summary, the addition of CaMnO_3_ had significant effects on the structural, magnetic, and electrical properties of BFO thin films. Magnetic investigations revealed that the magnetic characteristics of BFO were strongly influenced by CaMnO_3_, resulting in a ferromagnetic order at low temperatures and an antiferromagnetic order at room temperature. In the ferroelectric study, the 5 CaBFMO film displayed a pinched hysteresis loop, while the 10 CaBFMO film exhibited a well-saturated loop. Intriguingly, the polarization current curves for both films displayed the presence of four switching current peaks, characteristic of antiferroelectric-like behavior. It appears that the co-doping of Ca and Mn into the BFO matrix induced changes in the cation ordering at the A-/B-sites, leading to local structural heterogeneity that varied with the concentration of CaMnO_3_. Additionally, reversible resistive switching was observed in the 10 CaBFMO film when an electric field was applied. Overall, the co-doping of BFO with Ca and Mn (CaMnO_3_) shows promise for enhancing the functionalities of BFO materials, opening up opportunities for advanced applications and novel device technologies.

## Data Availability

Data are contained within the article.
